# HEARING LOSS AND FRAILTY: LONGITUDINAL ASSOCIATIONS

**DOI:** 10.1093/geroni/igac059.1161

**Published:** 2022-12-20

**Authors:** Nicholas Reed, Kening Jiang

**Affiliations:** Johns Hopkins Bloomberg School of Public Health, Baltimore, Maryland, United States; Johns Hopkins Cochlear Center for Hearing and Public Health, Baltimore, Maryland, United States

## Abstract

Hearing Loss (HL) is common among older adults and is associated with factors (e.g., walking speed and social isolation) that may mediate an association with frailty. In the National Health and Aging Trends Study (NHATS) 2011-2018 data, frailty was defined by the physical frailty phenotype composite (exhaustion, low physical activity, weakness, slowness, and shrinking) while HL was self-report. Among, 6897 baseline participants in 2011, 1132 (16%) were frail. In a cox proportional hazard model adjusted for demographic, socioeconomic, and clinical risk factors, HL (n=1607; Hazard Ratio[HR]=1.15; 95% Confidence Interval[CI]=1.00-1.32) was associated with higher risk of incident frailty relative to those with no HL (n=5290). In a generalized estimating equation longitudinal model (2011-2018), HL was associated with higher odds of frailty at baseline (Odds Ratio[OR]=1.52; 95%CI=1.49-1.96) and a 1.06 (95%CI=1.04-1.09) annual increase in odds of frailty. Future research should focus on mechanisms underlying association and determine the impact of HL treatment.

